# Modern Ophthalmology

**Published:** 1904-11

**Authors:** 


MODERN OPHTHALMOLOGY. A PRACTICAL TREATISE ON THE ANATOMY, PHYSIOLOGY, AND DISEASES OF THE EYE. By JAMES MOORE BALL, M.D., Professor of Ophthalmology in the Saint Louis College of Physicians and Surgeons. Octavo, pp. XXIV, 820, with 417 illustrations in the text and 21 colored plates. Philadelphia: F. A. Davis Company. 1904. (Price, cloth, $7.00; leather, $8.00.)

Ball's treatise is the latest candidate for professional favor in the department of ophthalmology. It enters the field in competition with many other works of acknowledged completeness and excellency, and to hold its own it must possess intrinsic merit and value, either equal to or excelling those already on the market. Our examination of it indicates that such undoubtedly is the case.

The aim of the author has been "to produce a work which shall teach and shall be valuable alike to the medical student, to the general practitioner, and to the specialist," and in so doing to "correctly represent the present advanced state of ophthalmic science and practice." At the outset, it may be doubted that the needs of the student, the general practitioner, and the specialist are altogether the same, and that any book can meet the necessarily dissimilar needs of the three different classes of readers. An attempt, therefore, to completely satisfy them in one treatise must be more or less unsuccessful. There is, however, a class of readers, who may look to such a common source of study, made up of the students who have an ambition to learn more than the mere elements of ophthalmology, the general practitioners who desire to find more information than is contained in the ordinary compend or manual, and the specialists who wish to review more or less fully the subject they have in hand. It is to this conglomerate class made up of the three, and animated even by varied motives that such a work as this is adapted. It is this class, too, and it is by no means a small one, that creates a demand for it. Whether such a demand is not already pretty well supplied is a question not to be discussed here. The author has expressed his opinion that there is at least room for it, by the fact of the publication of his book. It is not to be denied that there is always room for meritorious work in ophthalmology, and this treatise, like all others, must stand or fall on its own merits. There is certainly an appreciative public to which it appeals, and by satisfying its requirements it will live and succeed.

The work is divided into twenty-five chapters, and includes the whole subject of embryology, anatomy, physiology, and diseases of the eye, together with a chapter on hygiene of the eyes, and one on the preservation of eyeballs for demonstration and the methods employed in microscopic examination of the eye. While the work cannot be considered a composite one, the greater part being by Dr. Ball, himself, yet several important chapters are contributed by others. In arrangement of subjects, the author has adopted the anatomical classification, treating of glaucoma and sympathetic eye diseases after considering diseases of the vitreous humor, retina and optic nerve. Following diseases of the structures of the eyeball is a chapter on diseases of the orbit, by William T. Shoemaker; one on anomalies of the muscular apparatus, by William Zentmeyer; one by John T. Krall, on refraction; one by John T. Knipe, on the ocular manifestations of nervous diseases; one on hygiene of the eyes, by H. G. Goldberg, and finally, one on methods employed in microscopic examination of the eye, by W. E. Fischer.

Thus, it will be seen that the work extends over the whole range of ophthalmology. In dealing with the various subjects a completeness has been obtained that is very unusual. Scarcely any morbid condition of the eye, or any therapeutic measure of value has been omitted, and there are very few important facts, either in anatomy or physiology, which are not referred to or described. In short, nearly every subject has been brought well up to date. The author may not always have put the emphasis where some of his readers feel that it belongs, but in the main the descriptions are ample, the pathology is in accordance with the latest conclusions, and the therapeutics, both medical and surgical, is conservative, yet efficient, and includes the verified experiences of a wide range of contributors. The arrangement is systematic, and the style is clear without diffuseness.

Accompanying a text so comprehensively presented, and so clearly written, is a selection of illustrations of unusual merit and instructiveness. Many of them are new and original, and those in colors are unusually good. Those by the now famous painter of the fundus oculi, Margaretta Washington, of Philadelphia, are particularly commendable. All of them, however, so far as the printer's art permits, clearly and accurately represent the pathological conditions under consideration. Dr. Ball has produced a work of superior quality which deserves a wide circulation and careful study. A. A. H.

